# Human epidermal stem cell differentiation is modulated by specific lipid subspecies

**DOI:** 10.1073/pnas.2011310117

**Published:** 2020-08-25

**Authors:** Matteo Vietri Rudan, Ajay Mishra, Christian Klose, Ulrike S. Eggert, Fiona M. Watt

**Affiliations:** ^a^Centre for Stem Cells and Regenerative Medicine, King’s College London, SE1 9RT London, United Kingdom;; ^b^European Bioinformatics Institute, CB10 1SD Hinxton, United Kingdom;; ^c^Lipotype GmbH, 01307 Dresden, Germany;; ^d^Randall Centre for Cell and Molecular Biophysics, King’s College London, SE1 1UL London, United Kingdom

**Keywords:** lipids, epidermis, differentiation, keratinocytes, lipidomics

## Abstract

While the lipids of the outer layers of mammalian epidermis and their contribution to barrier formation have been extensively described, the role of individual lipid species in the onset of keratinocyte differentiation remains unknown. A lipidomic analysis of primary human keratinocytes revealed accumulation of numerous lipid species during suspension-induced differentiation. A small interfering RNA screen of 258 lipid-modifying enzymes identified two genes that on knockdown induced epidermal differentiation: *ELOVL1*, encoding elongation of very long-chain fatty acids protein 1, and *SLC27A1*, encoding fatty acid transport protein 1. By intersecting lipidomic datasets from suspension-induced differentiation and knockdown keratinocytes, we pinpointed candidate bioactive lipid subspecies as differentiation regulators. Several of these—ceramides and glucosylceramides—induced differentiation when added to primary keratinocytes in culture. Our results reveal the potential of lipid subspecies to regulate exit from the epidermal stem cell compartment.

## Significance

Cells generate a vast repertoire of lipid molecules whose functions are poorly understood. To investigate whether lipids can regulate cell fate decisions, we carried out a systematic lipidomic analysis and perturbation of lipid metabolism in cultured human epidermal keratinocytes, determining associations with the onset of differentiation. We identified individual lipid species that induced exit from the epidermal stem cell compartment. Our observations suggest that more research is warranted on the regulation of biological processes via lipid species, moving beyond the more conventional contribution of proteins and nucleic acids.

The skin is the largest organ in the human body, performing the primary functions of preventing water evaporation and protecting the internal organs from damaging external agents. The outermost part of the skin, the epidermis, is a stratified squamous epithelium formed predominantly by keratinocytes (1, 2). These cells migrate from the basal layer, home of the stem cell compartment, upward toward the surface of the skin through the spinous and granular layers to finally reach the cornified layer. During this migration, keratinocytes undergo a terminal differentiation process, accumulating transglutaminase-cross-linked proteins and secreting lamellar bodies enriched in highly hydrophobic lipid species. Ultimately, keratinocytes lose their nuclei and become corneocytes, held together by cross-linked sheets of apolar lipids in a structure that has been likened to bricks and mortar (1, 3).

Human epidermis can be reconstituted in culture, forming stratified sheets in which the stem cell compartment and key elements of the terminal differentiation process are preserved (1, 4). Clonal growth assays are used as a quantitative readout of stem cell abundance in cultures of human epidermal keratinocytes (4–6). Keratinocytes can be induced to undergo terminal differentiation in a near-synchronous manner when maintained as a single-cell suspension in medium containing methylcellulose (7, 8). The cells undergo a differentiation commitment phase after 4 h and increase expression of differentiation markers between 8 and 24 h (8, 9). While detailed transcriptomic and proteomic analysis of suspension-induced differentiation was carried out previously (9), little is known about changes in the lipid composition of keratinocytes as they exit the stem cell compartment.

Lipids are essential for the establishment of an efficient epidermal barrier (10). Alterations in the lipid composition of the cornified layer can lead to morphological defects in the epidermis and diseases such as ichthyoses (11–13). The predominant lipid species change across the different epidermal layers, with the basal and spinous layers enriched in more polar lipid classes such as phospholipids and sphingomyelins, the granular layers exhibiting higher levels of glycosylceramides and cholesterol sulfates, and the cornified layer mostly composed of cholesterol, fatty acids, and ceramides, particularly ω-acylceramides that are only found in the outermost epidermal layer (14). These differences are the cumulative result of keratinocyte lipid metabolism, sebaceous gland secretion, and microbial production (10, 12, 15).

Beyond their roles as structural components of cells and energy storage molecules, lipids function as bioactive compounds in a wide number of cellular processes. Posttranslational addition of lipid moieties to certain proteins is necessary for their function (16), and multiple classes of lipids are involved in endocrine (e.g., steroid hormones), paracrine (e.g., eicosanoids), or intracellular signaling (e.g., the phosphatidylinositol bisphosphate/diacylglycerol second-messenger system) (17). However, several other lipid classes, such as the sphingolipids ceramide and sphingosine-1-phosphate (18, 19), have signaling properties across multiple tissues, including the epidermis (20). Within each lipid class a cell produces a plethora of individual lipid species (i.e., carbon chain length, desaturation, and hydroxylation variants), whose importance is only beginning to be investigated (21, 22). Indeed, several studies suggest tight regulation and diverse functions for specific lipid molecular structures in a variety of cellular processes, ranging from cell division (23) to the innate immune response (24).

While the lipid composition of the outer layers of the epidermis has been well described, the potential role of individual lipid species in the early phases of keratinocyte differentiation remains to be investigated. Due to the high complexity of the lipid makeup of the epidermis and the limitations of methodologies available to manipulate lipids, we sought to approach this question in a more tractable system, namely, cultured primary human keratinocytes. We have used a combination of small interfering RNA (siRNA) screening and lipidomic analysis of primary human keratinocytes grown in culture. We find changes in sphingolipids and phospholipids associated with differentiation and show that several ceramide and glucosylceramide molecules can induce differentiation of cultured keratinocytes.

## Results

### The Lipid Composition of Keratinocytes Changes during Suspension-Induced Differentiation.

**Fig. 1.**
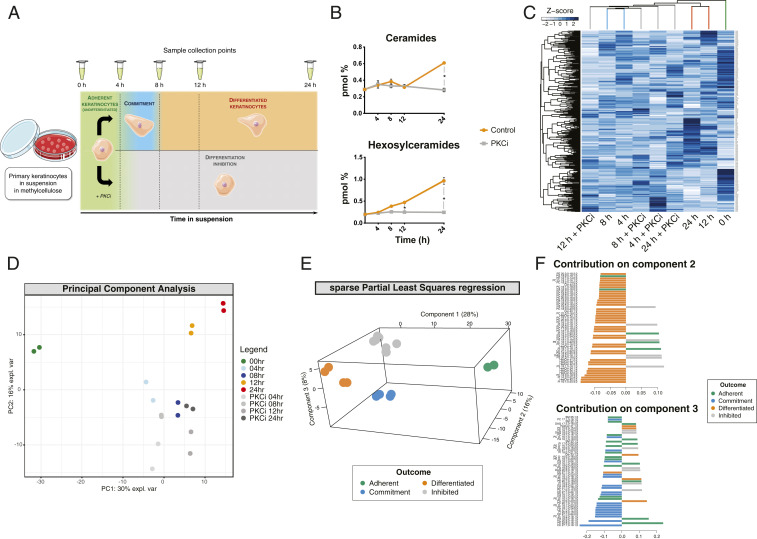
Keratinocyte lipid composition changes during suspension-induced differentiation. (*A*) Schematic overview of the experimental strategy and keratinocyte response to treatments (9). (*B*) Class-level variation of ceramides and hexosylceramides during suspension-induced keratinocyte differentiation, expressed as a percentage of the total sample lipids. Error bars indicate SDs. (*C*) Heat map representation and two-dimensional (2D) clustering of samples (Euclidean distance and complete-linkage clustering) based on the Z-scores of all lipid species (*y* axis) identified in the lipidomic analysis of suspension-induced keratinocyte differentiation; different time points/conditions are shown along the *x* axis; dendrogram branches are color-coded according to the schematic in *A*. (*D*) Sample variation along the first two principal components. (*E*) Separation along three components by sPLS after fitting the samples to the model shown in *A*. (*F*) Contribution of the 50 most discriminant lipid species to the separation along the second (*Upper* panel) and third (*Lower* panel) components of sPLS. Error bars indicate SDs; *P* values are calculated using multiple *t* tests with Holm–Sidak adjustment for multiple comparisons (*P < 0.05).

Single-cell suspension-induced differentiation of primary human keratinocytes can be blocked in the presence of a protein kinase C inhibitor (PKCi) (Fig. 1*A*) (9). To examine how lipids were affected in suspension culture, we analyzed samples using a shotgun lipidomics approach (25), comparing control and PKCi-treated cells (Dataset S1). Analysis of lipid changes at the class level showed significant accumulation of ceramides and hexosylceramides as keratinocytes underwent differentiation in suspension, mirroring the increase in these lipid classes that occurs during epidermal differentiation in vivo (14). This accumulation was blocked on inhibition of PKC (Fig. 1*B*). None of the other lipid classes showed such a marked change with time in suspension, although it was notable that several families were affected by PKC inhibition (*SI Appendix*, Fig. S1*A*). Unsupervised clustering based on lipid species abundance showed three main groups of samples: 1) those at the 0 h time point (adherent cells), 2) untreated cells suspended for 12 and 24 h (differentiated cells), and 3) untreated cells suspended for 4 and 8 h (committed cells) together with all of the PKCi-treated samples (Fig. 1*C*). Consistent with this, principal component analysis (PCA) separated the adherent cell sample from all of the others along the first component and the differentiated cells along the second, while the committed and inhibited cells could not be clearly separated (Fig. 1*D*).

In order to identify the most critical lipid species that accumulated during commitment and differentiation, the samples were first classified into four categories (“adherent,” “commitment,” “differentiated,” and “inhibited”) according to the model in Fig. 1*A* and subsequently examined using sparse partial least squares regression (sPLS) analysis to maximize separation between the sample groups, coupled with discriminant analysis (DA) to pinpoint the main lipid species contributing to such separation (26). The first and second components in the sPLS regression separated adherent and differentiated samples from the rest, respectively, while the samples undergoing commitment could be isolated along the third component (Fig. 1*E*). The contribution of lipid species to components 2 and 3 could therefore identify which discriminating molecules were enriched in the committed and differentiated samples, where a total of 145 lipids were found to accumulate (Fig. 1*F* and *SI Appendix*, Fig. S1*C* and Table S1).

These results show that during suspension-induced differentiation keratinocytes extensively change their lipid composition and accumulate a number of specific lipid species.

### Knockdown of Lipid-Modifying Enzymes Can Induce Differentiation.

Having identified a panel of lipids enriched during keratinocyte differentiation, we asked whether any of them regulated the onset of differentiation. Despite numerous advances, current methodologies do not allow direct and systematic manipulation of lipid molecular species (27). We therefore introduced perturbations in the lipid composition of adherent cultures of primary human keratinocytes by transfecting them with a panel of 258 siRNAs against lipid-modifying enzymes (23). The transfected cells were incubated under conditions (feeder-free keratinocyte serum-free medium [KSFM]) that would enrich for undifferentiated cells or were treated with medium supplemented with fetal bovine serum, which is known to stimulate accumulation of differentiated cells (28, 29). Immunofluorescence staining for involucrin, an early marker of differentiation in cultured keratinocytes (9, 30), was used as a readout of differentiation (Dataset S2).

**Fig. 2.**
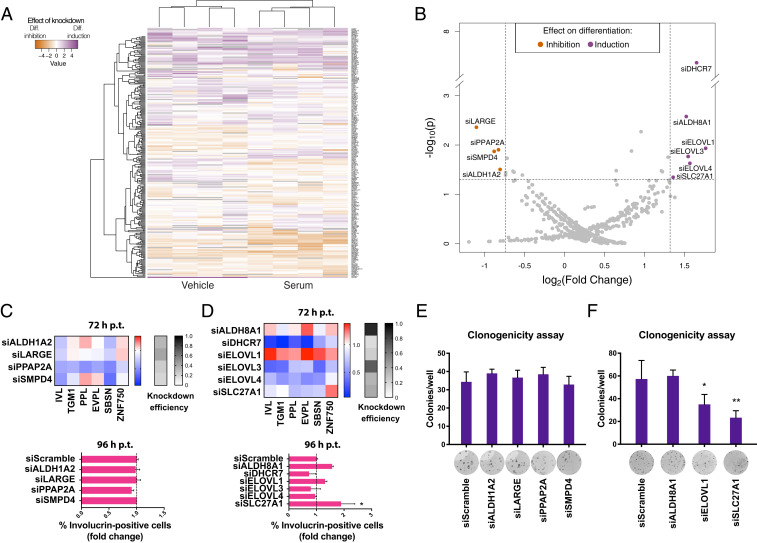
siRNA-mediated knockdown of lipid-modifying enzymes can affect keratinocyte differentiation. (*A*) Heat map representation of the Z-scores of involucrin levels in 258 lipid-modifying enzyme knockdowns (*y* axis) after 2D clustering of samples (*x* axis) (Euclidean distance and complete-linkage clustering). (*B*) Volcano plot of the screening results to identify hits based on statistical significance (*P* < 0.05) and fold change (FC) with respect to nontargeting siRNA controls included in each individual plate (FC < 0.6 for differentiation inhibition; FC > 2.5 for differentiation induction). Validation of differentiation-inhibiting (*C*) and differentiation-inducing (*D*) knockdowns by qPCR of differentiation markers (*Upper*) and immunofluorescence staining of involucrin (*Lower*); p.t.: posttransfection. Colony formation assay validation of differentiation-inhibiting (*E*) and differentiation-inducing (*F*) knockdowns with representative images for each siRNA. Error bars indicate SDs; *P* values are calculated using Dunnet’s multiple comparison test (*P < 0.05, **P < 0.01).

Modified Z-score transformation of the data (using the sample median and median absolute deviation) allowed pooling of both culture conditions in a single dataset. The siRNA screen yielded reproducible results, as indicated by the good correlation observed between each replicate and the mean of the quadruplicates (Pearson’s *r* ∼ 0.8; *SI Appendix*, Fig. S2*A*). No correlation was seen when plates containing different siRNAs were plotted against each other (*SI Appendix*, Fig. S2*B*), ruling out possible contributions of a sample’s position within a plate. siRNA against involucrin was used to estimate transfection efficiency and to validate the antibody (*SI Appendix*, Fig. S2*C*). Unsupervised two-way clustering of the screen results grouped together replicates of the same treatment but not related enzymes (Fig. 2*A*). Ten knockdowns elicited a response, with four inhibiting differentiation and six inducing it, based on statistical significance and fold change with respect to the average of control wells within the same plate (Fig. 2*B*).

The candidate enzymes were validated by confirming the effect of knockdown on involucrin protein expression and analyzing the transcription of a panel of additional differentiation markers in either differentiating (for the knockdowns that inhibited differentiation, Fig. 2*C*) or nondifferentiating conditions (for the knockdowns that induced differentiation, Fig. 2*D*). None of the candidate differentiation-inhibiting knockdowns significantly impacted the ability of keratinocytes to undergo differentiation, whereas the knockdown of three enzymes was indeed able to induce expression of involucrin and at least one other differentiation marker. We subsequently performed colony formation assays to further validate these effects, and in two cases (siELOVL1 and siSLC27A1) we were able to confirm a significant induction of keratinocyte differentiation on the basis of a reduction in colony formation, a surrogate of stem cell activity (9)(Fig. 2*F*). We also performed colony formation assays on the whole panel of candidate differentiation-inhibiting knockdowns but did not observe any significant effects (Fig. 2*E*). Deconvolution of the two validated siRNA pools did not suggest off-target effects (*SI Appendix*, Fig. S2*E*).

These results reveal that perturbation of lipid metabolism in primary human keratinocytes can interfere with terminal differentiation.

### Lipidomic Analysis Identifies Potential Lipid Regulators of Keratinocyte Differentiation.

When a lipid-modifying enzyme is down-regulated, it is difficult to predict how the lipid composition of the cell will be affected. This is because cellular lipid metabolism is extremely complex and redundant and many of the “building blocks” of complex lipid species are shared across multiple lipid classes (18). To accurately define the lipid species involved in regulating differentiation, we performed a comprehensive lipidomic analysis of cells transfected with siRNA targeting *ELOVL1* or *SLC27A1* or a nontargeting siRNA to control for the potential influence of transfection reagents (31). We collected samples at 24, 48, or 72 h posttransfection and performed lipidomics analysis on them (Dataset S3).

**Fig. 3.**
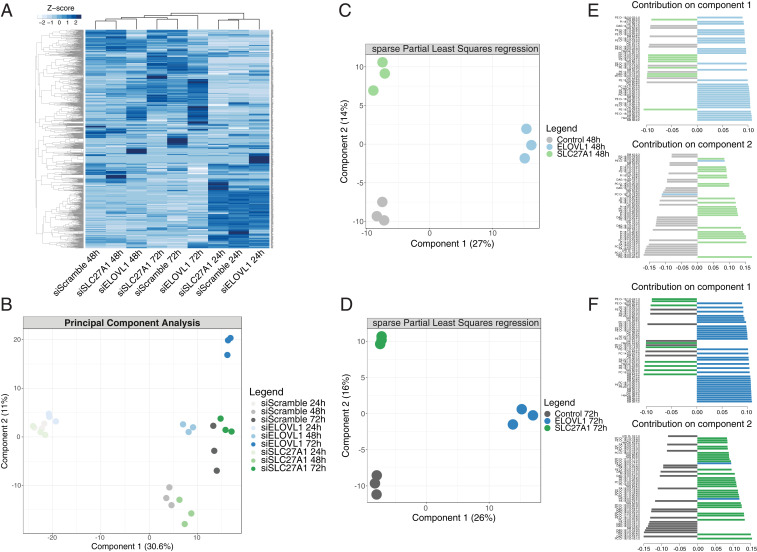
*ELOVL1* or *SLC27A1* knockdowns promote accumulation of specific lipid molecules. (*A*) Heat map representation and 2D clustering (Euclidean distance and complete-linkage clustering) of the Z-scores of all lipid species identified in the lipidomic analysis of differentiation-inducing knockdowns and control keratinocytes. (*B*) Sample variation along the first two principal components. (*C* and *D*) Sample separation along the first two components by sPLS of the 48 h (*C*) and 72 h (*D*) samples. (*E* and *F*) Contribution of the 50 most discriminant lipid species to the separation along the first and second components of sPLS for the 48 h (*E*) and 72 h (*F*) samples.

A class-level analysis of the lipidomics data did not show any obvious divergence in accumulation/depletion trends between the control and knocked-down samples (*SI Appendix*, Fig. S3*B*). When looking at the full panel of individual lipid species, unsupervised clustering of the samples (Fig. 3*A*) revealed that the main factor contributing to sample grouping was time, with cultured primary keratinocytes exhibiting a striking plasticity in their lipid composition, even in control conditions. Principal component analysis was consistent with this observation, in that the three different time points, irrespective of treatment, separated neatly along the first component (Fig. 3*B*). However, at 48 and 72 h the *ELOVL1* knockdown cells clustered separately from the other samples, indicating that the down-regulation of this enzyme caused a shift in the lipid makeup of the cells. Conversely, the siSLC27A1 samples were never clearly distinguishable from the nontargeting control samples.

### Specific Lipid Molecules Can Induce Keratinocyte Differentiation in Culture.

**Fig. 4.**
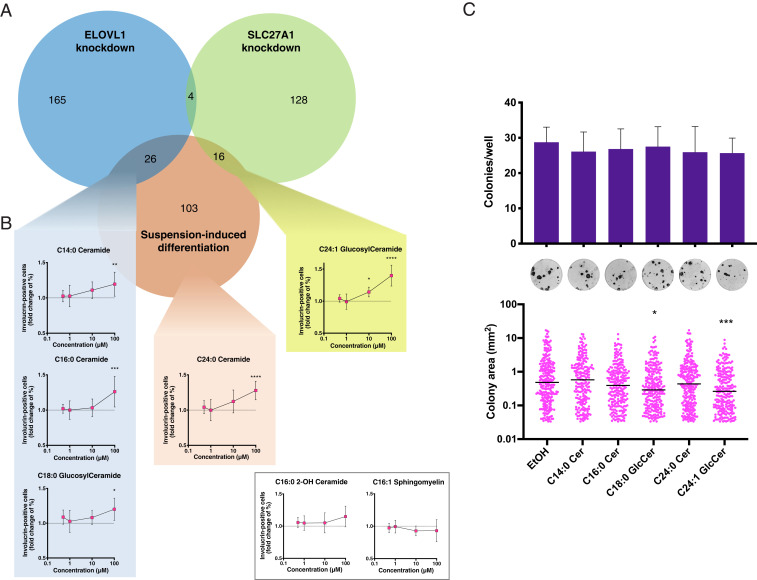
Specific lipid molecules can induce keratinocyte differentiation in culture. (*A*) Overlap between the discriminant lipid sets enriched during ELOVL1 knockdown, SLC27A1 knockdown, or suspension-induced keratinocyte differentiation. (*B*) Keratinocyte response to different doses of ceramides and glucosylceramides identified in the ELOVL1 intersection enriched lipid set (teal), in the SLC27A1 intersection lipid set (yellow), in the suspension-induced differentiation enriched lipid set (orange), or in no set (white box). Error bars indicate SDs, *P* values are calculated using one-way ANOVA with Dunnett’s multiple comparison test (*P < 0.05, **P < 0.01, ***P < 0.001, ****P < 0.0001), comparing each lipid treatment to vehicle-treated cells (1% ethanol, represented by a dashed line in the graphs). (*C*) Effect of bioactive lipid treatments on the colony formation ability of keratinocytes. Shown are the total number of colonies per well (dark purple bars) as well as the number of abortive colonies (magenta bars). Representative images for each treatment are shown below the bar plot. Error bars indicate SDs, *P* values are calculated using one-way ANOVA with Dunnett’s multiple comparison test (colony number) or the Kruskal–Wallis test with Dunn’s multiple comparison test (colony size) (*P < 0.05, ***P < 0.001).

To identify the critical lipid species that accumulated in the knockdown cells, we conducted a sPLS-DA analysis on the 48 and 72 h samples separately (Fig. 3 *C* and *D*). The analysis identified 195 unique discriminant lipid species enriched in siELOVL1 cells at either time point. Interestingly, despite the similarity to the controls, the analysis was also able to separate with good accuracy the siSLC27A1 samples and allowed us to pinpoint 148 discriminant lipid species that accumulated in these cells (Fig. 3 *E* and *F* and *SI Appendix*, Fig. S3 *C* and *D* and Tables S2 and S3). We next compared the lipids enriched in committed and differentiated cells (Fig. 1) with the ones that accumulated in *ELOVL1* or *SLC27A1* knocked-down keratinocytes to see if there were any common species. Remarkably, these two independent experimental approaches, which stimulated differentiation in completely different ways, yielded an overlap of 26 and 16 candidate bioactive lipid species in the case of the *ELOVL1* and *SLC27A1* knockdown, respectively, that could potentially function as differentiation inducers (“intersection sets”; Fig. 4*A*). The overlapping lipid species were sphingolipids (12/42), more specifically ceramides (6/42) and hexosylceramides (6/42), and glycerophospholipids (30/42), with the most represented types being phosphatidylcholines (8/42) and phosphatidylserines (7/42). The fatty acid moieties present had chain lengths ranging between 14 and 24 carbon atoms, containing up to four unsaturations (Table 1).

**Table 1.** Lipid molecules enriched both upon ELOVL1 or SLC27A1 knockdown and upon suspension-induced differentiation of primary human keratinocytes

Knockdown	Lipid species*	Max fold change^†^	Tested lipid	Differentiation induction
siELOVL1	Ceramide – 32:1:2 (C14:0 Cer)	1.7 (72 h)	C14:0 ceramide	Yes
siELOVL1	Ceramide – 34:1:2 (C16:0 Cer)	1.7 (48 h)	C16:0 ceramide	Yes
siELOVL1	Ceramide – 34:2:2 (C16:1 Cer)	1.5 (72 h)	—	—
siELOVL1	Ceramide – 38:1:2 (C20:0 Cer)	8.3 (72 h)	—	—
siELOVL1	Ceramide – 40:1:2 (C22:0 Cer)	1.3 (72 h)	C24:0 ceramide (accumulated in suspension-differentiated cells)	Yes
siELOVL1	Ceramide – 40:2:2 (C22:1 Cer)	1.7 (72 h)	—	—
siELOVL1	Hexosylceramide – 34:1:2 (C16:0 HexCer)	1.5 (72 h)	—	—
siELOVL1	Hexosylceramide – 36:1:2 (C18:0 HexCer)	Accumulated^‡^ (48 and 72 h)	C18:0 glucosylceramide	Yes
siELOVL1	Hexosylceramide – 38:1:2 (C20:0 HexCer)	Accumulated^‡^ (48 and 72 h)	—	—
siELOVL1	Hexosylceramide – 40:1:2 (C22:0 HexCer)	3 (72 h)	—	—
siELOVL1	Hexosylceramide – 40:2:2 (C22:1 HexCer)	Accumulated^‡^ (48 and 72 h)		
siELOVL1	Phosphatidylcholine – 17:0:0;17:1:0	3.7 (48 h)	—	—
siELOVL1	Phosphatidylcholine – 18:0:0;18:1:0	1.1 (72 h)	—	—
siELOVL1	Phosphatidylcholine – 18:0:0;18:2:0	2 (72 h)	—	—
siELOVL1	Phosphatidylcholine – 18:1:0;18:1:0	1.2 (72 h)	—	—
siELOVL1	Phosphatidylcholine – 18:1:0;18:2:0	1.1 (48 h)	—	—
siELOVL1	Phosphatidylcholine – 18:2:0;18:2:0	1.3 (48 h)	—	—
siELOVL1	Phosphatidylethanolamine ether – 18:1:0;18:1:0	1.05 (48 h)		
siELOVL1	Phosphatidylethanolamine ether – 18:1:0;20:3:0	1.2 (72 h)	—	—
siELOVL1	Phosphatidylethanolamine ether – 18:1:0;20:4:0	1.2 (72 h)	—	—
siELOVL1	Phosphatidylinositol – 16:1:0;20:4:0	Accumulated^‡^ (48 h)	—	—
siELOVL1	Phosphatidylserine – 14:0:0;18:1:0	1.8 (48 h)	—	—
siELOVL1	Phosphatidylserine – 16:1:0;20:0:0	3.2 (48 h)		
siELOVL1	Phosphatidylserine – 18:1:0;20:0:0	1.9 (48 h)	—	—
siELOVL1	Phosphatidylserine – 18:2:0;20:0:0	Accumulated^‡^ (48 h)	—	—
siELOVL1	Phosphatidylserine – 18:2:0;22:1:0	3.7 (72 h)	—	—
siSLC27A1	Hexosylceramide – 42:2:2 (C24:1 HexCer)	1.1 (72 h)	C24:1(Δ9) glucosylceramide	Yes
siSLC27A1	Phosphatidic acid – 16:0:0;18:2:0	3.2 (48 h)	—	—
siSLC27A1	Phosphatidylcholine – 16:1:0;16:1:0	1.1 (48 h)	—	—
siSLC27A1	Phosphatidylcholine – 18:0:0;20:3:0	1.3 (48 h)	—	—
siSLC27A1	Phosphatidylcholine ether – 16:1:0;16:1:0	1.2 (72 h)	—	—
siSLC27A1	Phosphatidylcholine ether – 16:1:0;20:4:0	4.2 (72 h)	—	—
siSLC27A1	Phosphatidylcholine ether – 18:1:0;16:1:0	1.3 (72 h)	—	—
siSLC27A1	Phosphatidylcholine ether – 18:1:0;20:4:0	1.1 (72 h)	—	—
siSLC27A1	Phosphatidylethanolamine ether – 16:1:0;20:3:0	1.1 (72 h)	—	—
siSLC27A1	Phosphatidylglycerol – 16:1:0;18:0:0	Accumulated^‡^ (48 h)	—	—
siSLC27A1	Phosphatidylglycerol – 18:2:0;18:2:0	2.4 (72 h)	—	—
siSLC27A1	Phosphatidylglycerol – 18:2:0;20:3:0	7 (72 h)	—	—
siSLC27A1	Phosphatidylinositol – 16:2:0;18:0:0	Accumulated^‡^ (48 h)	—	—
siSLC27A1	Phosphatidylinositol – 18:2:0;20:2:0	4 (48 h)	—	—
siSLC27A1	Phosphatidylserine – 16:1:0;18:1:0	1.3 (48 h)	—	—
siSLC27A1	Phosphatidylserine – 18:0:0;19:1:0	Accumulated^‡^ (48 h)	—	—

* Lipids are annotated as “Class – Carbon chain length: Unsaturations: Hydroxylations.” For sphingolipids, the numbers refer to the total in the molecule; for phospholipids, individual lengths of the two fatty acid moieties are reported. The alternative notation used for sphingolipids refers to the carbon chain length of their fatty acid moiety and its number of unsaturations.

^†^ Fold changes are calculated based on the percentage picomole relative to total sample lipids.

^‡^ A numeric value could not be calculated due to undetectable levels in the control.

We sought next to verify whether any of the species identified could stimulate keratinocytes to undergo differentiation. Hexosylceramides can have a glucose or a galactose as their head group, but we assumed them to be glucosylceramides due to the established presence and importance of this lipid class in the epidermis (14, 32). We tested two ceramides and two glucosylceramides from our intersection sets and included one more ceramide species that differed from a candidate molecule by having a chain two carbon atoms longer and still appeared in the set enriched during suspension-induced differentiation. As a negative control, we employed one ceramide and one sphingomyelin species that did not appear in either of our enriched lipid sets. Notably, the negative control ceramide had the same chemical structure as the lipids in the intersection sets, with the exception of bearing an α-hydroxylated fatty acid moiety.

We incubated cultured primary human keratinocytes for 48 h with increasing concentrations of our candidate bioactive lipids or the control lipids. At the highest concentration tested (100 µM) all candidate lipids, but not the control lipids, produced a significant increase in involucrin-expressing cells (Fig. 4*B*). None of the bioactive lipids were toxic, as assessed by cleaved caspase 3 staining (*SI Appendix*, Fig. S4). To further validate the effect of the lipids, we tested whether they could affect the ability of keratinocytes to form colonies. Incubation with the lipids for 48 h reduced the average number of colonies formed in all conditions, albeit not significantly. Addition of either candidate glucosylceramide significantly decreased colony size (Fig. 4*C*).

## Discussion

Our studies show that in addition to forming the epidermal barrier, acting as intracellular signaling molecules, and modulating the skin microbiome (15), epidermal lipids can regulate exit from the epidermal stem cell compartment. By integrating lipidomic datasets with an siRNA screen of lipid modifying enzymes, we identified *ELOVL1* and *SLC27A1* as genes that, when knocked down, caused keratinocytes to undergo differentiation. The knockdown of *ELOVL1* and, to a lesser extent, *SLC27A1* caused a shift in the lipid composition of keratinocytes, and introduction of individual ceramides and glucosylceramides mimicked the ability of the knockdowns to promote differentiation. It should be noted that our analysis did not cover the full gamut of lipid classes, lacking, for example, eicosanoids and fatty acids. It is therefore possible that additional lipid species also participate in the regulation of keratinocyte differentiation.

ELOVL1 catalyzes the elongation of saturated and monounsaturated C20 to C26 acyl-CoAs. ELOVL1 activity is linked to the production of sphingolipids as it is regulated by ceramide synthase (CerS) enzymes (33). *Elovl1* knockout mice die shortly after birth due to skin barrier deficiencies caused by the impaired formation of lipid lamellae and defective desquamation (34). These mice are depleted of ≥C26 ceramides and, similar to our results, accumulate ≤C24 ceramides but do not present any major alterations in keratinocyte differentiation (34). Recent studies have described a mutation in *ELOVL1* in humans that causes similar alterations in the lipid profile and is associated with dry skin and ichthyotic keratoderma, pointing to abnormal differentiation and therefore supporting our findings (35, 36).

FATP1, encoded by the *SLC27A1* gene, is a fatty acid transporter that possesses a very long chain fatty acyl-CoA synthetase activity (37). It is expressed in the basal layer of adult human epidermis (38). FATP1 can be localized in the endoplasmic reticulum or in the plasma membrane (39), which might indicate a role in the intracellular distribution of lipids rather than their abundance. This would be consistent with *SLC27A1* knockdown affecting differentiation without strongly perturbating the overall lipid composition of keratinocytes. *Slc27a1*^−/−^ mice do not exhibit skin phenotypes (39), and a loss-of-function mutation in humans linked to Melkersson–Rosenthal syndrome does not produce an epidermal phenotype (40). This potentially indicates the existence of compensatory mechanisms to maintain skin homeostasis in vivo.

A common set of individual lipid molecules was enriched in cells stimulated to differentiate by suspension culture or by either *ELOVL1* or *SLC27A1* knockdown. The bioactive lipid mediators we tested, ceramides and glucosylceramides, are known to be important in the biology of the upper epidermal layers (32). Our results are in line with an early report that a mixture of glucosylceramides can induce differentiation in fetal rat skin keratinocytes (41) and shed light on the individual molecular species responsible. A more recent study (42) detailed lipid profile changes and alterations in keratinocyte differentiation in patients with autosomal recessive congenital ichthyosis with mutations in the ceramide synthase 3 (CerS3) gene. These patients showed a deficiency in longer-chain (≥C26) ceramides. Interestingly, CerS3 can influence the activity of ELOVL1 by enhancing further elongation of C24 fatty acyl-CoA to C26/C28 fatty acyl-CoA (34). These observations point to a key role of the ≤C24/≥C26 ceramide (and possibly hexosylceramide) ratio in regulating keratinocyte differentiation. Building on these studies, we now define a number of specific lipid subtypes that are able to influence the fate of keratinocytes. Notably, these molecules must operate within restricted chemical boundaries, as the addition of structurally similar lipids to the growth medium was unable to significantly affect keratinocyte differentiation.

The amplitude of the effect of the bioactive lipids we describe may depend on several factors, such as how efficiently the lipid molecules are delivered to the appropriate subcellular destination or a dependency of the biological effect on the contextual lipid (and perhaps protein) composition of the cell (24). This latter concept is consistent with the idea that lipids operate by affecting the functionality of whole assemblies of molecular partners within their localized environment, which makes their effects challenging to evaluate using the more linear approaches that are commonly employed when studying proteins (27).

Our data offer insights into the influence of lipid composition on the modulation of cellular behavior and represent a starting platform to study the role of lipid species in the cellular biology of the epidermis at the molecular level. In this respect, while the regulatory potential of individual lipid subspecies on cellular function is largely unexplored, there are a number of interesting possibilities. The Wnt pathway is a key regulator of stem cells in many, if not all, tissues, and Wnts require lipid modifications for activity (43). Therefore, their action could be affected by direct interactions with key lipids within membranes or by changes in their local environment due to lipid variation. Lipid composition can affect the stiffness and mechanosensitivity of plasma membranes (44–46), and differences in the stiffness of epidermal stem cells and neighboring basal cells in culture were recently reported (47).

Whereas keratinocytes undergoing suspension-induced differentiation lack cell–cell contacts, the siRNA experiments and lipid treatments were performed on adherent cells which have intercellular junctions. In the principal component analysis of the lipidomic data the majority of the variance (component 1) separated cells that had been disaggregated and immediately lysed (time 0) from cells that had been maintained in suspension (Fig. 1*D*), suggesting that intercellular adhesion may affect lipid composition. Within the epidermis, basal keratinocytes delaminate as they move into the suprabasal layers during differentiation, and lipids could potentially play a role in this process by altering the properties of the cell membrane or modulating the activity of junctional proteins (48, 49). For example, the localization of the desmosomal cadherin desmoglein-1 to specific glycosylceramide-enriched membrane domains is essential for keratinocyte delamination during the early phases of differentiation (49). Further analysis will be required to discover how the cellular- and tissue-level architecture of the epidermis is impacted by the lipid changes we have identified. In addition, lipids are important in inflammation (24, 50) and could, potentially, mediate aspects of epidermal immune signaling.

In conclusion, we have systematically shown that the lipid composition of human keratinocytes critically impacts their differentiation, down to the level of individual lipid subspecies. Our comprehensive approach has allowed us to identify a set of specific lipid molecules that induce differentiation when added to primary keratinocytes in culture. Since it is not technically feasible to specifically target the basal layer of the epidermis for lipid delivery, either in in vitro reconstituted skin or in vivo, the physiological relevance of our findings remains to be explored fully. It also remains technically challenging to dissect the exact mechanisms through which the lipid subspecies identified are operating. Nevertheless, the results we report corroborate a number of recent studies (23, 24) in underlining the importance and largely unexplored regulatory potential of individual lipid subspecies in cellular function and lay the foundation for further exploration of the role of lipids in epidermal differentiation.

## Materials and Methods

For a summary of materials and resources used, see *SI Appendix*, Table S4.

### Primary Human Keratinocyte Culture.

Primary male human keratinocytes (strain Km) isolated from neonatal foreskin were cultured at 37 °C on mitotically inactivated 3T3-J2 cells in complete F12/adenine/Dulbecco’s modified eagle medium (FAD) medium, containing one part Ham’s F-12, three parts Dulbecco’s modified eagle medium, 100 µM adenine, 10% (vol/vol) fetal bovine serum (FBS), 0.5 μg/mL hydrocortisone, 5 μg/mL insulin, 0.1 nM cholera toxin, and 10 ng/mL epidermal growth factor (EGF), as described previously (9). Primary keratinocytes were used in experiments at passages 4–7. For knockdown or overexpression experiments, keratinocytes were grown in KSFM medium (Gibco) supplemented with 0.15 ng/mL EGF and 30 μg/mL bovine pituitary extract. When subculturing or seeding cells for an experiment, we filtered the disaggregated keratinocytes through a 40 µm nylon strainer to remove cell clumps and large differentiated keratinocytes.

### siRNA Screening and Transfection.

We transfected primary human keratinocytes with a custom siRNA library targeting 258 lipid biosynthetic enzymes purchased from Dharmacon (siGENOME pools) (23). In addition, every plate contained two negative nontargeting siRNA control wells, two no-siRNA wells, and two involucrin-targeting siRNA wells. Nontargeting and no-siRNA wells were used as negative controls, while involucrin-targeting wells were used as positive controls for the estimation of transfection efficiency and validation of antibody specificity. The screening was performed in quadruplicate in two different culture conditions. Reverse transfection of siRNAs was performed in accordance with the transfection reagent manufacturer’s instructions. siRNAs were diluted in KSFM medium and arrayed in 96-well plates (Greiner µClear). The siRNAs were incubated for 20 min with 10 µL of INTERFERin-HTS transfection reagent (PolyPlus Transfection) diluted 1/200 in water. Approximately 8,000 keratinocytes were then seeded in every well in 125 µL complete KSFM medium. Final volume was 175 µL per well, and the final siRNA concentration was 30 nM. Four hours after transfection, the medium was changed to fresh complete KSFM with added 1% penicillin/streptomycin. Two days after transfection, half the plates were switched to complete KSFM + penicillin/streptomycin supplemented with 10% FBS to induce keratinocyte differentiation; in the remaining plates, the medium was refreshed. Ninety-six hours after transfection, the plates were washed once with phosphate-buffered saline (PBS) and fixed with 4% paraformaldehyde incubated for 10 min at room temperature. Plates were then permeabilized by incubating with 0.2% Triton-X-100 in PBS for 5 min at room temperature and incubated with blocking buffer (10% FBS, 0.25% fish skin gelatin in PBS) for 1 h and stained overnight at +4 °C with the primary antibody anti-involucrin (SY3 or SY7 clones) diluted to 1 µg/mL in blocking buffer. Plates were then washed three times with PBS, stained with the secondary antibody Alexa Fluor 555 donkey anti-mouse (Thermo Fisher Scientific), the nuclear dye DRAQ5 (abcam), and Alexa Fluor 647 Phalloidin (Thermo Fisher Scientific) at a concentration of 1 µg/mL, 10 µM, and 12.6 nM in blocking buffer, respectively. Secondary stains were incubated for 2 h at room temperature protected from light, and plates were subsequently washed three times with PBS before being imaged using the Perkin-Elmer Operetta High-Content Imaging System. For validation experiments, a similar protocol was performed, but using the ONTARGET plus siRNA series (Horizon Discovery).

### Immunofluorescence.

Antibody staining of cells was performed in the same way as described in *siRNA Screening and Transfection*. Plates were washed once with PBS and fixed with 4% paraformaldehyde incubated for 10 min at room temperature. Plates were then permeabilized by incubating with 0.2% Triton-X-100 in PBS for 5 min at room temperature, incubated with blocking buffer (10% FBS, 0.25% fish skin gelatin in PBS) for 1 h and stained overnight at +4 °C with the primary antibody anti-involucrin (SY3 or SY7 clones) or anti-cleaved caspase 3 (Asp175) (Cell Signaling catalog no. 9661) diluted to 1 µg/mL or according to manufacturer’s instructions in blocking buffer. Plates were then washed three times with PBS, stained with the secondary antibodies Alexa Fluor 555 donkey anti-mouse (Thermo Fisher Scientific) and/or Alexa Fluor 488 donkey anti-mouse (Thermo Fisher Scientific) at 1 µg/mL, the nuclear dye DRAQ5 (abcam) at 10 µM, and Alexa Fluor 647 Phalloidin (Thermo Fisher Scientific) at 12.6 nM in blocking buffer. Secondary stains were incubated for 2 h at room temperature protected from light, and plates were finally washed three times with PBS before being imaged using the Perkin-Elmer Operetta High-Content Imaging System.

### High-Content Imaging Analysis.

Images acquired with the Perkin-Elmer Operetta High-Content Imaging System were analyzed using custom algorithms in the Perkin-Elmer Harmony high-content analysis software package (*SI Appendix*, Fig. S2*D*). Nuclei were initially defined using the DRAQ5 channel; small (<2,000 µm^2^) and highly irregular (roundness < 0.6) nuclei were excluded from the analysis. To minimize the misattribution of the cytoplasm areas, the level of cytoplasmic staining was inferred from the fluorescence intensity in a ring around the nucleus, as involucrin staining was homogeneous throughout the cytoplasm. Terminally differentiating cells were identified by manual thresholding of the involucrin perinuclear fluorescence intensity. The complete Harmony image analysis sequence has been deposited in Open Science Framework (https://osf.io/wf7v2/?view_only=4b1f32c0e8d14c3fa13b95d51247e503). Data from both screening conditions (Dataset S2) was transformed using modified Z-scores (calculated with the sample median and median absolute deviation) and compiled into a single data set. Fold changes were calculated with respect to negative control samples present in the same plate as each test sample.

### RNA Isolation, Complementary DNA Synthesis, and qPCR.

RNA was extracted from all samples using the RNeasy Mini kit (Qiagen) according to the manufacturer’s instructions and subsequently reverse transcribed using the QuantiTect Reverse Transcription kit (Qiagen) according to the manufacturer’s instructions. The complementary DNA was diluted to 5 ng/µL, and specific targets were amplified by qPCR using the Fast SYBR Green Master Mix (Thermo Fisher Scientific). Primer details can be found in *SI Appendix*, Table S4. The amplification program used was as follows: 3 min incubation at 95 °C followed by 40 cycles of denaturation for 3 s at 95 °C and annealing/extension for 25 s at 60 °C; melting curve analysis was also performed to ensure amplifications were specific. Expression of all targets was normalized against the expression of three reference genes (RPL13A, ATP5B, and TBP) and against control sample expression (ΔΔC_q_).

### Suspension-Induced Keratinocyte Differentiation.

Keratinocytes were differentiated in suspension as described previously (8, 9). Preconfluent cultures were disaggregated in trypsin/ethylenediaminetetraacetic acid and resuspended at a concentration of 10^5^ cells/mL in medium containing 1.45% methylcellulose, supplemented with either 5 µM GF109203X (Tocris) to inhibit PKC or dimethylsulphoxide as a control. Aliquots were plated in six-well plates coated with 0.4% polyhydroxyethylmethacrylate; this ensured that there was no cell–substratum adhesion. The suspended cells were subsequently incubated at 37 °C. At each collection time point, the methylcellulose was diluted with PBS, and the cells were recovered by centrifugation. The experiment was performed in duplicate.

### Clonogenicity Assays.

Five hundred keratinocytes were plated on a 3T3 feeder layer per well of a six-well dish. After 12 d, feeders were removed, and keratinocyte colonies were fixed in 4% paraformaldehyde (Sigma) for 10 min then stained with 1% rhodanile blue (1:1 mixture of rhodamine B and Nile blue A [Acros Organics]). Colony number and size were scored using ImageJ.

### Lipid Treatment of Cultured Primary Human Keratinocytes.

All lipid molecules were purchased from Avanti Polar Lipids (see *SI Appendix*, Table S4) in powder form. The lipids were dissolved in ethanol at a stock concentration of 10 mM and stored at −20 °C. In each experiment, 15,000 keratinocytes were seeded per well of a 96-well plate and incubated overnight before adding the lipids. Upon treatment, the stock solutions were heated to 37 °C, diluted to the indicated concentrations in culture medium preheated to 37 °C, and finally added to the monolayers. Vehicle concentration was 1%, equal to the level present in the highest lipid concentration.

### Lipid Extraction for Mass Spectrometry (MS) Lipidomics.

MS-based lipidomics was performed by Lipotype GmbH (51). A two-step chloroform/methanol procedure was used to extract the lipids (52), and samples were spiked with an internal lipid standard mixture. This standard comprised cardiolipin 16:1/15:0/15:0/15:0 (CL), ceramide 18:1;2/17:0 (Cer), diacylglycerol 17:0/17:0 (DAG), hexosylceramide 18:1;2/12:0 (HexCer), lysophosphatidate 17:0 (LPA), lysophosphatidylcholine 12:0 (LPC), lysophosphatidylethanolamine 17:1 (LPE), lysophosphatidylglycerol 17:1 (LPG), lysophosphatidylinositol 17:1 (LPI), lysophosphatidylserine 17:1 (LPS), phosphatidate 17:0/17:0 (PA), phosphatidylcholine 17:0/17:0 (PC), phosphatidylethanolamine 17:0/17:0 (PE), phosphatidylglycerol 17:0/17:0 (PG), phosphatidylinositol 16:0/16:0 (PI), phosphatidylserine 17:0/17:0 (PS), cholesterol ester 20:0 (CE), sphingomyelin 18:1;2/12:0;0 (SM), triacylglycerol 17:0/17:0/17:0 (TAG), and cholesterol D6 (Chol). The organic phase was dried in a speed vacuum concentrator. The dried extract was resuspended in 7.5 mM ammonium acetate in chloroform/methanol/propanol (1:2:4, vol:vol:vol), dried again, and then suspended in a 33% ethanol solution of methylamine in chloroform/methanol (0.003:5:1, vol:vol:vol). Liquid handling was performed on the Hamilton Robotics STARlet robotic platform with the Anti Droplet Control feature.

### MS Data Acquisition.

Sample analysis (53) was performed on a Q Exactive mass spectrometer (Thermo Scientific) with a TriVersa NanoMate ion source (Advion Biosciences). Analysis was performed in positive and negative ion modes (resolution = 280,000 at *m/z* = 200 for MS and resolution = 17,500 at *m/z *= 200 for tandem MS [MSMS], where *m/z* indicates the mass-to-charge ratio). The trigger for MSMS was an inclusion list of MS mass ranges scanned in 1-Da increments. MS and MSMS data were combined to monitor CE, DAG, and TAG ions as ammonium adducts; PC and PC O- as acetate adducts; and CL, PA, PE, PE O-, PG, PI, and PS as deprotonated anions. MS alone was used to monitor LPA, LPE, LPE O-, LPI, and LPS as deprotonated anions; Cer, HexCer, SM, LPC, and LPC O- as acetate adducts; and cholesterol as the ammonium adduct of an acetylated derivative.

### Data Analysis and Postprocessing.

Data were analyzed with in-house-developed lipid identification software based on LipidXplorer (53). Data postprocessing and normalization were performed using an in-house-developed data management system. Lipids identified with a signal-to-noise ratio of >5 and a signal intensity fivefold higher than in corresponding blank samples were analyzed further.

### Downstream Analysis of Lipidomics Data.

Lipid amounts from the lipidomics experiments were normalized by the total amount of lipids in each sample. The samples were compared by unsupervised hierarchical clustering using the R heatmap.2 function (package Rplots) with default settings. Samples in the siRNA knockdown time course were analyzed by PCA using the mixOmics package (v6.10.9) (54) with parameter scale = T after removal of near-zero variance predictors. To maximize separation between the samples at 48 and 72 h posttransfection and identify discriminating lipid species, we employed sPLS-DA using the mixOmics package with the following parameters: ncomp = 3, keepX = rep(250,3), near.zero.var = T. Samples in the suspension-induced differentiation time course were analyzed by PCA using the mixOmics package with parameters ncomp = 3, scale = T after removal of near-zero variance predictors. To maximize separation between the samples and identify discriminating lipid species, we classified the samples according to the model detailed in Fig. 2 (9) and employed sPLS-DA using the mixOmics package with the following parameters: ncomp = 3, keepX = rep(250,3), near.zero.var = T. Discriminating lipids in the siRNA knockdown time course experiment were identified as lipids enriched in siELOVL1 that were contributing to the separation from siScramble along component 1 in the sPLS-DA at 48 or 72 h posttransfection (loading > 0.01) and lipids enriched in siELOVL1 or siSLC27A1 that were contributing to the separation from siScramble along component 2 at 48 or 72 h posttransfection (loading > 0.01). Enrichment and fold change were determined by comparing the means of the replicates of each sample. Discriminating lipids in the suspension-induced differentiation time course experiment were identified as lipids enriched in the “differentiated” class samples (see *Results*) that were contributing to the separation from all other sample classes along component 2 in the sPLS-DA (loading < −0.01) or lipids that were enriched in “commitment” class samples (see *Results*) that were contributing to the separation from all other sample classes along component 3 in the sPLS-DA (loading < −0.01). Enrichment was determined by comparing the median of each sample class.

### Data Analysis.

Data are presented as mean and standard deviation (SD). Statistical analysis was performed using Microsoft Excel and GraphPad Prism 8.0. Normality was assessed based on quantile–quantile plot inspection. See figure legends for details of the statistical tests used. For the siRNA screening, *n* = 4 replicates (independent transfections). For validation experiments, *n* ≥ 2. For the knockdown time course lipidomics, *n* = 3 replicates (independent transfections). For the suspension-induced differentiation lipidomics, *n* = 2 replicates. For lipid-induced involucrin expression, *n* ≥ 6 independent treatments. For lipid-induced apoptosis induction, *n* ≥ 3 independent treatments. For colony formation assays, *n* ≥ 2 hexaplicates of independent transfections or *n* = 12 independent treatments.

## Supplementary Material

Supplementary File

Supplementary File

Supplementary File

Supplementary File

## Data Availability

All study data are included in the article and *SI Appendix*.
