# Supplementary material for: Human epidermal stem cell differentiation is modulated by specific lipid subspecies
Source: Proc Natl Acad Sci U S A. 2020 Aug 25;117(36):22173–82. doi: 10.1073/pnas.2011310117 (PMC7486749; doi:10.1073/pnas.2011310117)
Supplement: Supplementary File [file pnas.2011310117.sapp.pdf]

PNAS
www.pnas.org

Supplementary Information for

Human epidermal stem cell differentiation is modulated by specific lipid sub-species

Matteo Vietri Rudan, Ajay Mishra, Christian Klose, Ulrike S. Eggert and Fiona M. Watt

Corresponding author: Fiona M. Watt
Email: fiona.watt@kcl.ac.uk

This PDF file includes:

Figures S1 to S4
Tables S1 to S4
Legends for Datasets S1 to S3

Other supplementary materials for this manuscript include the following:

Datasets S1 to S3

www.pnas.org/cgi/doi/10.1073/pnas.2011310117

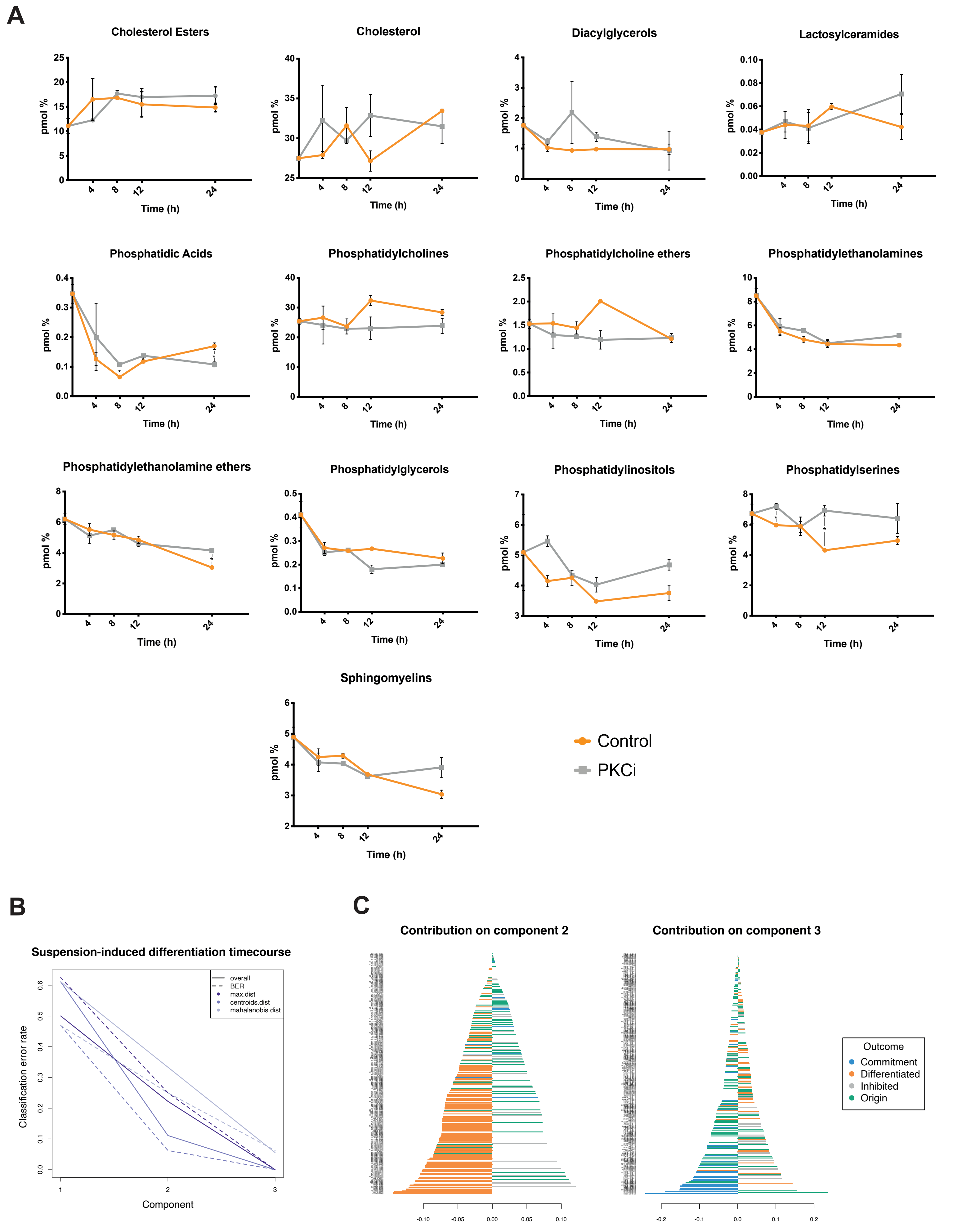

Fig. S1. Lipidomic characterisation of suspension-induced keratinocyte differentiation. (*A*) Variation in the overall levels of multiple lipid classes over the differentiation time course. The y-axes represent the percentage picomole relative to the total amount of lipids in each sample. Error bars indicate standard deviations, p-values are calculated using multiple t tests with Holm-Sidak adjustment for multiple comparisons (*p<0.05). (*B*) Leave-one-out cross validation of the sPLS-DA model. (*C*) Discriminant lipid species found by sPLS-DA analysis separating samples along the second (left graph) and third (right graph) component.

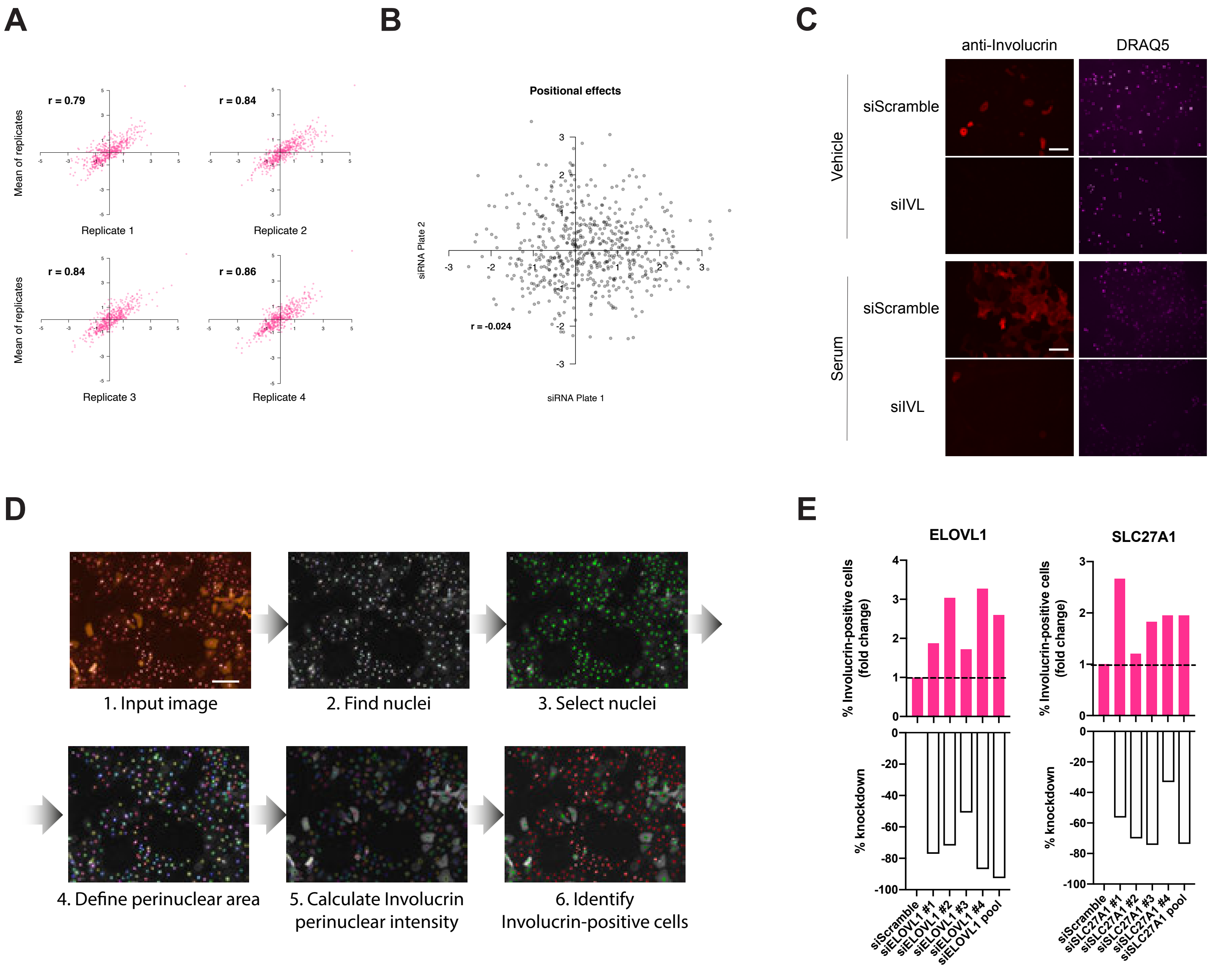

Fig. S2. Screen of lipid-modifying enzyme knockdown effects on keratinocyte differentiation. (*A*) Correlation between the Z-scores of the percent Involucrin-positive cells in each screening replicate against the mean of the quadruplicates (r = Pearson correlation coefficient). (*B*) Correlation between the Z-scores of the percent Involucrin-positive cells in two different plates to assess the influence of well position (r = Pearson correlation coefficient). (*C*) Representative images of Involucrin staining in control and Involucrin-knockdown wells in the absence (top panels) or the presence (bottom panels) of serum as a differentiation inducer. Scale bar: 200 μm. (*D*) Image analysis pipeline in Harmony software for identification of Involucrin-positive cells. Input images (1) are segmented to identify nuclei (2) that are subsequently filtered for size (> 2000 μm2) and roundness (> 0.6) (3). A ring-shaped area around each nucleus is then defined as a surrogate for the cytoplasm and Involucrin fluorescence intensity is calculated in this area (4). A manually curated threshold is applied to call Involucrin-positive cells (5). Scale bar: 200 μm. (*E*) Deconvolution of screening hits siRNA. Knockdown levels and Involucrin-positive cell % increase over control for each siRNA are shown.

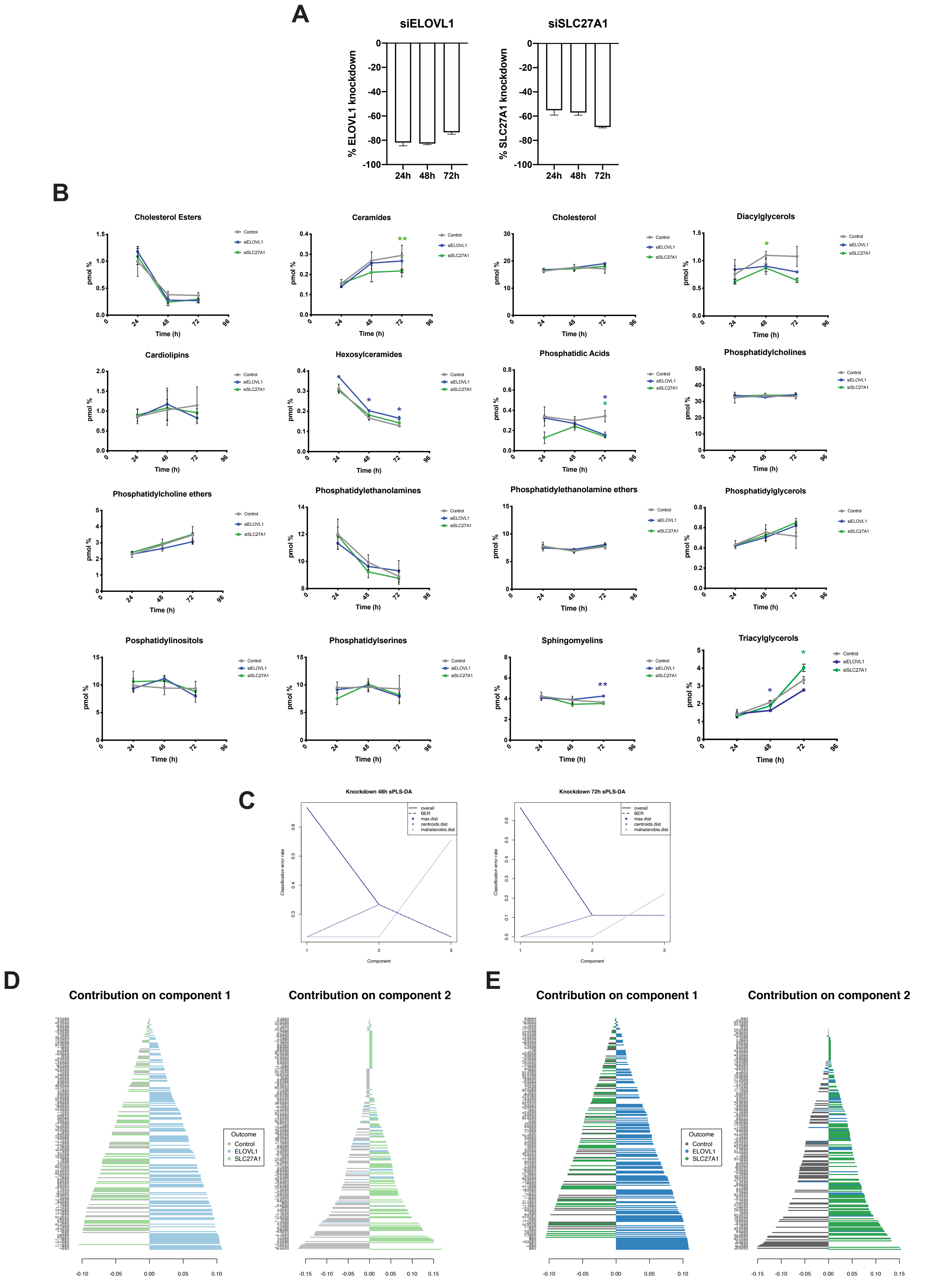

Fig. S3. Lipidomic characterisation of screen hits. (*A*) Knockdown efficiency of ELOVL1 in siELOVL1 samples (left) and SLC27A1 in siSLC27A1 samples (right) at different times after transfection. (*B*) Variation in the overall levels of multiple lipid classes over the knockdown time course. The y-axes represent the percentage picomole relative to the total amount of lipids in each sample. Error bars indicate standard deviations, p-values are calculated using two-way ANOVA with Dunnett's multiple comparisons test against the control (*p<0.05, **p<0.01, asterisks are colour-coded according to the sample they refer to). (*C*) Leave-one-out cross validation of the sPLS-DA model at 48 h (left) and 72 h (right) post-transfection. (*D*) Discriminant lipid species found by sPLS-DA analysis separating samples along the first and second component at 48 h post-transfection. (*E*) Discriminant lipid species found by sPLS-DA analysis separating samples along the first and second component at 72 h post-transfection.

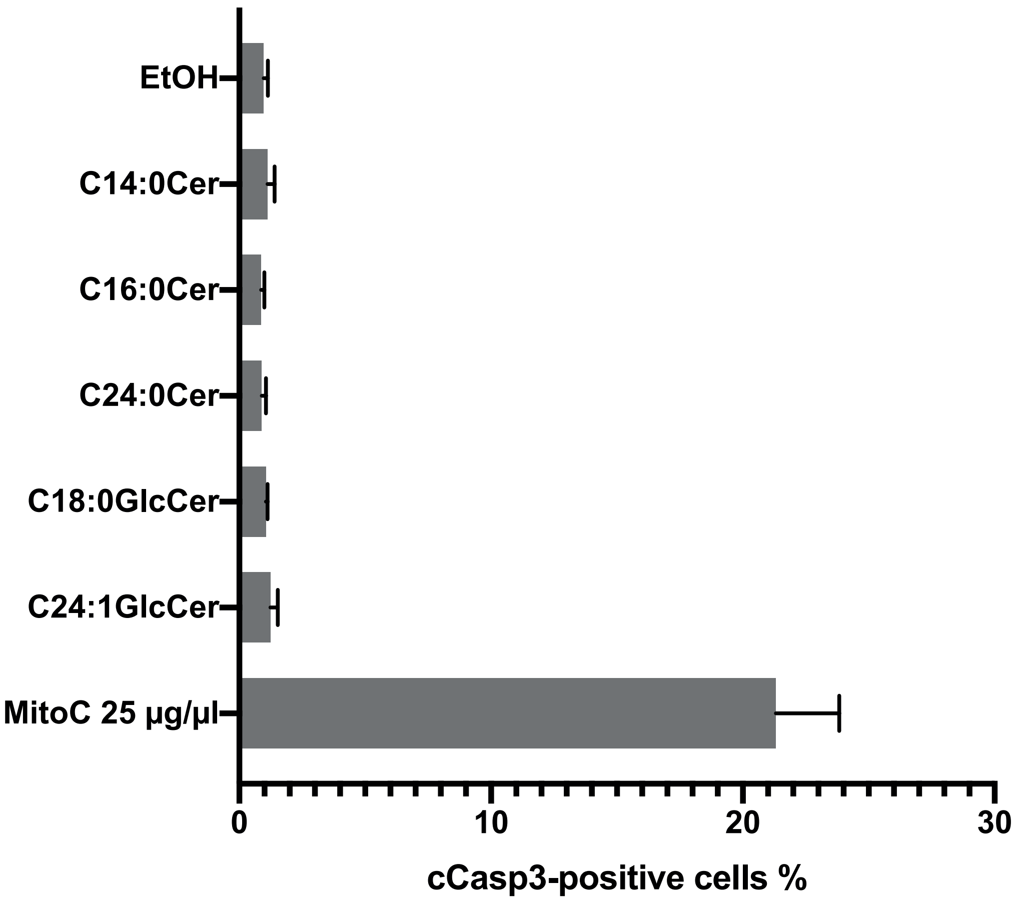

Fig. S4. Effect of lipid treatments on apoptosis. Percentage of cleaved caspase-3 (cCasp3) positive cells after treatment with 100 μM of the indicated lipids or with 25 μg/ml Mitomycin C for 48 h. Cleaved caspase-3 levels were assessed by immunofluorescence staining.

Table S1. Lipids enriched during commitment or differentiation

Ceramide - 31:4:2	PhosphatidylCholine - 16:0:0;20:5:0	PhosphatidylCholine - 22:3:0;16:0:0
Ceramide - 32:1:2	PhosphatidylCholine - 16:1:0;14:0:0	PhosphatidylCholine - 22:3:0;18:0:0
Ceramide - 33:1:2	PhosphatidylCholine - 16:1:0;16:1:0	PhosphatidylCholine - 22:4:0;18:1:0
Ceramide - 34:1:2	PhosphatidylCholine - 17:0:0;16:0:0	PhosphatidylCholine - 22:5:0;16:0:0
Ceramide - 34:2:2	PhosphatidylCholine - 17:0:0;16:1:0	PhosphatidylCholine - 22:5:0;18:1:0
Ceramide - 36:1:2	PhosphatidylCholine - 17:0:0;17:1:0	PhosphatidylCholine Ether - 14:1:0;16:0:0
Ceramide - 36:2:2	PhosphatidylCholine - 17:0:0;18:2:0	PhosphatidylCholine Ether - 16:0:0;16:1:0
Ceramide - 38:1:2	PhosphatidylCholine - 17:1:0;16:0:0	PhosphatidylCholine Ether - 16:0:0;20:4:0
Ceramide - 39:1:2	PhosphatidylCholine - 17:1:0;16:1:0	PhosphatidylCholine Ether - 16:1:0;14:0:0
Ceramide - 40:1:2	PhosphatidylCholine - 18:0:0;15:0:0	PhosphatidylCholine Ether - 16:1:0;16:1:0
Ceramide - 40:2:2	PhosphatidylCholine - 18:0:0;18:1:0	PhosphatidylCholine Ether - 16:1:0;20:4:0
Ceramide - 41:1:2	PhosphatidylCholine - 18:0:0;18:2:0	PhosphatidylCholine Ether - 16:2:0;16:1:0
Ceramide - 41:2:2	PhosphatidylCholine - 18:0:0;18:3:0	PhosphatidylCholine Ether - 18:1:0;16:1:0
Ceramide - 42:1:2	PhosphatidylCholine - 18:0:0;20:3:0	PhosphatidylCholine Ether - 18:1:0;18:1:0
Ceramide - 42:2:2	PhosphatidylCholine - 18:0:0;20:4:0	PhosphatidylCholine Ether - 18:1:0;20:4:0
Ceramide - 42:3:2	PhosphatidylCholine - 18:0:0;20:5:0	PhosphatidylEthanolamine - 18:0:0;20:4:0
Cholesterol Esther - 27:1:0;14:0:0	PhosphatidylCholine - 18:1:0;15:0:0	PhosphatidylEthanolamine - 20:3:0;17:0:0
Cholesterol Esther - 27:1:0;14:1:0	PhosphatidylCholine - 18:1:0;16:0:0	PhosphatidylEthanolamine - 20:3:0;18:2:0
Cholesterol Esther - 27:1:0;16:1:0	PhosphatidylCholine - 18:1:0;16:2:0	PhosphatidylEthanolamine - 22:3:0;16:0:0
Cholesterol Esther - 27:1:0;17:0:0	PhosphatidylCholine - 18:1:0;17:0:0	PhosphatidylEthanolamine - 22:4:0;18:0:0
Cholesterol Esther - 27:1:0;18:0:0	PhosphatidylCholine - 18:1:0;18:1:0	PhosphatidylEthanolamine Ether - 16:1:0;20:3:0
Cholesterol Esther - 27:1:0;18:1:0	PhosphatidylCholine - 18:1:0;18:2:0	PhosphatidylEthanolamine Ether - 18:1:0;18:1:0
Cholesterol Esther - 27:1:0;20:2:0	PhosphatidylCholine - 18:1:0;18:3:0	PhosphatidylEthanolamine Ether - 18:1:0;20:3:0
Cholesterol Esther - 27:1:0;20:3:0	PhosphatidylCholine - 18:1:0;20:4:0	PhosphatidylEthanolamine Ether - 18:1:0;20:4:0
Cholesterol Esther - 27:1:0;22:5:0	PhosphatidylCholine - 18:1:0;22:6:0	PhosphatidylEthanolamine Ether - 18:2:0;20:4:0
Cholesterol Esther - 27:1:0;24:0:0	PhosphatidylCholine - 18:2:0;14:0:0	PhosphatidylGlycerol - 16:0:0;18:2:0
Cholesterol Esther - 27:1:0;24:1:0	PhosphatidylCholine - 18:2:0;15:0:0	PhosphatidylGlycerol - 16:1:0;18:0:0
Cholesterol Esther - 27:2:0;20:0:0	PhosphatidylCholine - 18:2:0;16:0:0	PhosphatidylGlycerol - 18:2:0;18:2:0
Diacylglycerol - 18:0:0;16:0:0	PhosphatidylCholine - 18:2:0;16:1:0	PhosphatidylGlycerol - 18:2:0;20:3:0
HexCeramide - 34:0:2	PhosphatidylCholine - 18:2:0;18:2:0	PhosphatidylInositol - 16:0:0;22:4:0
HexCeramide - 34:1:2	PhosphatidylCholine - 19:0:0;18:1:0	PhosphatidylInositol - 16:1:0;20:4:0
HexCeramide - 35:1:2	PhosphatidylCholine - 19:1:0;18:1:0	PhosphatidylInositol - 16:2:0;18:0:0
HexCeramide - 36:1:2	PhosphatidylCholine - 19:1:0;18:2:0	PhosphatidylInositol - 18:0:0;22:4:0
HexCeramide - 38:1:2	PhosphatidylCholine - 19:2:0;16:0:0	PhosphatidylInositol - 18:2:0;20:2:0
HexCeramide - 40:0:2	PhosphatidylCholine - 20:1:0;17:0:0	PhosphatidylInositol - 19:2:0;18:0:0
HexCeramide - 40:1:2	PhosphatidylCholine - 20:1:0;18:0:0	PhosphatidylInositol - 20:5:0;16:0:0
HexCeramide - 40:2:2	PhosphatidylCholine - 20:1:0;18:1:0	PhosphatidylSerine - 14:0:0;18:1:0
HexCeramide - 41:1:2	PhosphatidylCholine - 20:1:0;18:2:0	PhosphatidylSerine - 16:1:0;18:1:0
HexCeramide - 41:2:2	PhosphatidylCholine - 20:2:0;16:0:0	PhosphatidylSerine - 16:1:0;20:0:0
HexCeramide - 42:0:2	PhosphatidylCholine - 20:2:0;16:1:0	PhosphatidylSerine - 18:0:0;19:1:0
HexCeramide - 42:0:3	PhosphatidylCholine - 20:2:0;18:1:0	PhosphatidylSerine - 18:0:0;22:0:0
HexCeramide - 42:1:2	PhosphatidylCholine - 20:2:0;18:2:0	PhosphatidylSerine - 18:1:0;20:0:0
HexCeramide - 42:1:3	PhosphatidylCholine - 20:3:0;16:0:0	PhosphatidylSerine - 18:1:0;24:1:0
HexCeramide - 42:2:2	PhosphatidylCholine - 20:3:0;16:1:0	PhosphatidylSerine - 18:2:0;20:0:0
HexCeramide - 42:2:3	PhosphatidylCholine - 20:3:0;18:1:0	PhosphatidylSerine - 18:2:0;22:0:0
Phosphatidic Acid - 16:0:0;18:2:0	PhosphatidylCholine - 20:3:0;18:2:0	PhosphatidylSerine - 18:2:0;22:1:0
PhosphatidylCholine - 16:0:0;14:3:0	PhosphatidylCholine - 20:3:0;20:3:0	PhosphatidylSerine - 18:2:0;24:1:0
PhosphatidylCholine - 16:0:0;16:2:0	PhosphatidylCholine - 20:4:0;16:0:0	
PhosphatidylCholine - 16:0:0;18:3:0	PhosphatidylCholine - 20:4:0;20:4:0	

Table S2. Lipids enriched in siELOVL1 keratinocytes

Cardiolipin - 72:4:0	PhosphatidylCholine Ether - 18:1:0;15:0:0	PhosphatidylEthanolamine Ether - 18:2:0;20:0:0
Ceramide - 32:1:2	PhosphatidylCholine Ether - 18:1:0;18:0:0	PhosphatidylEthanolamine Ether - 18:2:0;20:1:0
Ceramide - 32:2:2	PhosphatidylCholine Ether - 18:1:0;20:1:0	PhosphatidylEthanolamine Ether - 18:2:0;20:2:0
Ceramide - 34:1:2	PhosphatidylCholine Ether - 18:1:0;20:3:0	PhosphatidylEthanolamine Ether - 18:2:0;20:3:0
Ceramide - 34:2:2	PhosphatidylEthanolamine - 16:0:0;20:1:0	PhosphatidylEthanolamine Ether - 18:2:0;20:4:0
Ceramide - 38:1:2	PhosphatidylEthanolamine - 16:0:0;22:1:0	PhosphatidylEthanolamine Ether - 18:2:0;22:1:0
Ceramide - 38:2:2	PhosphatidylEthanolamine - 16:1:0;19:1:0	PhosphatidylEthanolamine Ether - 18:2:0;22:2:0
Ceramide - 40:1:2	PhosphatidylEthanolamine - 16:1:0;20:1:0	PhosphatidylEthanolamine Ether - 18:2:0;22:3:0
Ceramide - 40:2:2	PhosphatidylEthanolamine - 16:1:0;20:2:0	PhosphatidylEthanolamine Ether - 18:2:0;22:4:0
Cholesterol	PhosphatidylEthanolamine - 16:1:0;22:2:0	PhosphatidylEthanolamine Ether - 18:2:0;22:5:0
Diacylglycerol - 16:1:0;20:1:0	PhosphatidylEthanolamine - 16:1:0;22:5:0	PhosphatidylEthanolamine Ether - 18:2:0;22:6:0
Diacylglycerol - 18:1:0;20:1:0	PhosphatidylEthanolamine - 17:1:0;20:1:0	PhosphatidylGlycerol - 16:1:0;20:1:0
HexosylCeramide - 34:1:2	PhosphatidylEthanolamine - 18:0:0;20:2:0	PhosphatidylGlycerol - 18:1:0;18:1:0
HexosylCeramide - 36:1:2	PhosphatidylEthanolamine - 18:0:0;20:3:0	PhosphatidylGlycerol - 18:1:0;20:1:0
HexosylCeramide - 38:1:2	PhosphatidylEthanolamine - 18:0:0;22:2:0	PhosphatidylGlycerol - 18:1:0;20:2:0
HexosylCeramide - 40:1:2	PhosphatidylEthanolamine - 18:0:0;22:3:0	PhosphatidylGlycerol - 18:2:0;20:1:0
HexosylCeramide - 40:2:2	PhosphatidylEthanolamine - 18:0:0;22:4:0	PhosphatidylGlycerol - 18:2:0;20:2:0
LysoPhosphatidylCholine - 16:0:0	PhosphatidylEthanolamine - 18:1:0;20:1:0	PhosphatidylInositol - 16:0:0;22:1:0
LysoPhosphatidylCholine - 18:0:0	PhosphatidylEthanolamine - 18:1:0;20:2:0	PhosphatidylInositol - 16:1:0;18:2:0
LysoPhosphatidylEthanolamine - 18:1:0	PhosphatidylEthanolamine - 18:1:0;22:1:0	PhosphatidylInositol - 16:1:0;20:1:0
LysoPhosphatidylEthanolamine - 20:1:0	PhosphatidylEthanolamine - 18:1:0;22:2:0	PhosphatidylInositol - 16:1:0;20:4:0
LysoPhosphatidylEthanolamine - 20:3:0	PhosphatidylEthanolamine - 18:1:0;22:3:0	PhosphatidylInositol - 18:0:0;20:4:0
LysoPhosphatidylEthanolamine - 20:4:0	PhosphatidylEthanolamine - 18:1:0;22:4:0	PhosphatidylInositol - 18:1:0;18:1:0
PhosphatidylCholine - 14:0:0;19:1:0	PhosphatidylEthanolamine - 18:2:0;20:1:0	PhosphatidylInositol - 18:1:0;20:1:0
PhosphatidylCholine - 14:0:0;20:1:0	PhosphatidylEthanolamine - 18:2:0;20:2:0	PhosphatidylInositol - 18:2:0;18:2:0
PhosphatidylCholine - 14:0:0;20:2:0	PhosphatidylEthanolamine - 20:1:0;20:1:0	PhosphatidylInositol - 18:2:0;20:1:0
PhosphatidylCholine - 14:0:0;22:1:0	PhosphatidylEthanolamine - 20:1:0;20:2:0	PhosphatidylInositol - 20:1:0;20:2:0
PhosphatidylCholine - 15:0:0;17:0:0	PhosphatidylEthanolamine - 20:1:0;20:4:0	PhosphatidylInositol - 20:1:0;20:4:0
PhosphatidylCholine - 15:0:0;17:1:0	PhosphatidylEthanolamine - 20:2:0;20:2:0	PhosphatidylSerine - 14:0:0;18:1:0
PhosphatidylCholine - 15:0:0;20:1:0	PhosphatidylEthanolamine Ether - 16:0:0;18:1:0	PhosphatidylSerine - 16:0:0;20:1:0
PhosphatidylCholine - 16:0:0;17:1:0	PhosphatidylEthanolamine Ether - 16:0:0;20:1:0	PhosphatidylSerine - 16:0:0;20:2:0
PhosphatidylCholine - 16:0:0;19:1:0	PhosphatidylEthanolamine Ether - 16:0:0;20:2:0	PhosphatidylSerine - 16:0:0;22:1:0
PhosphatidylCholine - 16:0:0;20:1:0	PhosphatidylEthanolamine Ether - 16:0:0;20:3:0	PhosphatidylSerine - 16:1:0;20:0:0
PhosphatidylCholine - 16:0:0;20:2:0	PhosphatidylEthanolamine Ether - 16:0:0;20:4:0	PhosphatidylSerine - 16:1:0;20:1:0
PhosphatidylCholine - 16:1:0;18:2:0	PhosphatidylEthanolamine Ether - 16:0:0;22:3:0	PhosphatidylSerine - 16:1:0;20:2:0
PhosphatidylCholine - 16:1:0;19:1:0	PhosphatidylEthanolamine Ether - 16:0:0;22:5:0	PhosphatidylSerine - 16:1:0;22:1:0
PhosphatidylCholine - 16:1:0;20:1:0	PhosphatidylEthanolamine Ether - 16:1:0;19:1:0	PhosphatidylSerine - 17:1:0;20:1:0
PhosphatidylCholine - 16:1:0;20:2:0	PhosphatidylEthanolamine Ether - 16:1:0;20:1:0	PhosphatidylSerine - 18:0:0;20:0:0
PhosphatidylCholine - 16:2:0;18:1:0	PhosphatidylEthanolamine Ether - 16:1:0;20:2:0	PhosphatidylSerine - 18:0:0;20:1:0
PhosphatidylCholine - 17:0:0;17:1:0	PhosphatidylEthanolamine Ether - 16:1:0;21:3:0	PhosphatidylSerine - 18:0:0;20:2:0
PhosphatidylCholine - 17:1:0;17:1:0	PhosphatidylEthanolamine Ether - 16:1:0;22:3:0	PhosphatidylSerine - 18:0:0;22:4:0
PhosphatidylCholine - 17:1:0;18:0:0	PhosphatidylEthanolamine Ether - 16:2:0;20:1:0	PhosphatidylSerine - 18:1:0;20:0:0
PhosphatidylCholine - 17:1:0;18:1:0	PhosphatidylEthanolamine Ether - 17:1:0;19:1:0	PhosphatidylSerine - 18:1:0;20:1:0
PhosphatidylCholine - 17:1:0;20:1:0	PhosphatidylEthanolamine Ether - 17:1:0;20:2:0	PhosphatidylSerine - 18:1:0;21:1:0
PhosphatidylCholine - 18:0:0;18:1:0	PhosphatidylEthanolamine Ether - 17:1:0;20:3:0	PhosphatidylSerine - 18:1:0;22:1:0
PhosphatidylCholine - 18:0:0;18:2:0	PhosphatidylEthanolamine Ether - 18:0:0;18:1:0	PhosphatidylSerine - 18:1:0;22:3:0
PhosphatidylCholine - 18:0:0;20:2:0	PhosphatidylEthanolamine Ether - 18:0:0;20:1:0	PhosphatidylSerine - 18:2:0;20:0:0
PhosphatidylCholine - 18:1:0;18:1:0	PhosphatidylEthanolamine Ether - 18:0:0;20:2:0	PhosphatidylSerine - 18:2:0;20:1:0
PhosphatidylCholine - 18:1:0;18:2:0	PhosphatidylEthanolamine Ether - 18:0:0;20:3:0	PhosphatidylSerine - 18:2:0;22:1:0
PhosphatidylCholine - 18:1:0;19:1:0	PhosphatidylEthanolamine Ether - 18:0:0;20:4:0	PhosphatidylSerine - 20:1:0;20:1:0
PhosphatidylCholine - 18:1:0;20:1:0	PhosphatidylEthanolamine Ether - 18:1:0;16:0:0	PhosphatidylSerine - 20:1:0;20:3:0
PhosphatidylCholine - 18:1:0;20:2:0	PhosphatidylEthanolamine Ether - 18:1:0;18:1:0	Sphingomyelin - 32:0:2
PhosphatidylCholine - 18:1:0;22:2:0	PhosphatidylEthanolamine Ether - 18:1:0;20:1:0	Sphingomyelin - 32:1:2
PhosphatidylCholine - 18:2:0;18:2:0	PhosphatidylEthanolamine Ether - 18:1:0;20:2:0	Sphingomyelin - 32:2:2
PhosphatidylCholine - 18:2:0;20:1:0	PhosphatidylEthanolamine Ether - 18:1:0;20:3:0	Sphingomyelin - 34:1:2
PhosphatidylCholine - 18:2:0;20:2:0	PhosphatidylEthanolamine Ether - 18:1:0;22:1:0	Sphingomyelin - 34:1:3
PhosphatidylCholine - 20:1:0;20:1:0	PhosphatidylEthanolamine Ether - 18:1:0;22:2:0	Sphingomyelin - 34:2:2
PhosphatidylCholine - 20:1:0;20:2:0	PhosphatidylEthanolamine Ether - 18:1:0;22:3:0	Sphingomyelin - 36:1:2
PhosphatidylCholine Ether - 16:0:0;20:1:0	PhosphatidylEthanolamine Ether - 18:2:0;12:0:0	Sphingomyelin - 36:2:2
PhosphatidylCholine Ether - 16:1:0;17:0:0	PhosphatidylEthanolamine Ether - 18:2:0;16:0:0	Sphingomyelin - 38:1:2
PhosphatidylCholine Ether - 16:1:0;20:1:0	PhosphatidylEthanolamine Ether - 18:2:0;16:2:0	Sphingomyelin - 38:2:2
PhosphatidylCholine Ether - 16:1:0;20:3:0	PhosphatidylEthanolamine Ether - 18:2:0;17:1:0	Sphingomyelin - 40:1:2
PhosphatidylCholine Ether - 17:0:0;19:1:0	PhosphatidylEthanolamine Ether - 18:2:0;18:1:0	Sphingomyelin - 40:2:2
PhosphatidylCholine Ether - 18:0:0;18:1:0	PhosphatidylEthanolamine Ether - 18:2:0;18:2:0	Triacylglycerol - 56:3:0
PhosphatidylCholine Ether - 18:0:0;20:2:0	PhosphatidylEthanolamine Ether - 18:2:0;19:2:0	Triacylglycerol - 56:4:0

Table S3. Lipids enriched in siSLC27A1 keratinocytes

Cardiolipin - 64:4:0	PhosphatidylCholine Ether - 18:1:0;20:2:0	PhosphatidylGlycerol - 18:2:0;18:2:0
Cardiolipin - 66:2:0	PhosphatidylCholine Ether - 18:1:0;20:4:0	PhosphatidylGlycerol - 18:2:0;20:3:0
Cardiolipin - 66:4:0	PhosphatidylCholine Ether - 18:2:0;14:0:0	PhosphatidylInositol - 14:0:0;18:0:0
Cardiolipin - 68:6:0	PhosphatidylCholine Ether - 18:2:0;16:0:0	PhosphatidylInositol - 14:0:0;18:2:0
Cardiolipin - 72:7:0	PhosphatidylCholine Ether - 18:2:0;16:1:0	PhosphatidylInositol - 14:1:0;18:0:0
Diacylglycerol - 14:0:0;16:1:0	PhosphatidylCholine Ether - 18:2:0;20:1:0	PhosphatidylInositol - 16:0:0;16:1:0
Diacylglycerol - 14:1:0;16:0:0	PhosphatidylCholine Ether - 18:2:0;20:2:0	PhosphatidylInositol - 16:0:0;18:2:0
HexosylCeramide - 42:2:2	PhosphatidylCholine Ether - 18:2:0;20:3:0	PhosphatidylInositol - 16:0:0;20:2:0
LysoPhosphatidylInositol - 20:4:0	PhosphatidylEthanolamine - 12:0:0;18:1:0	PhosphatidylInositol - 16:1:0;18:1:0
Phosphatidic Acid - 16:0:0;18:2:0	PhosphatidylEthanolamine - 14:0:0;16:0:0	PhosphatidylInositol - 16:1:0;19:1:0
Phosphatidic Acid - 17:0:0;17:1:0	PhosphatidylEthanolamine - 14:0:0;16:1:0	PhosphatidylInositol - 16:1:0;20:2:0
PhosphatidylCholine - 14:0:0;14:0:0	PhosphatidylEthanolamine - 14:0:0;18:2:0	PhosphatidylInositol - 16:1:0;20:3:0
PhosphatidylCholine - 14:0:0;18:2:0	PhosphatidylEthanolamine - 16:0:0;20:4:0	PhosphatidylInositol - 16:2:0;18:0:0
PhosphatidylCholine - 15:0:0;16:0:0	PhosphatidylEthanolamine - 16:1:0;18:2:0	PhosphatidylInositol - 17:0:0;18:2:0
PhosphatidylCholine - 15:0:0;22:1:0	PhosphatidylEthanolamine - 16:1:0;20:3:0	PhosphatidylInositol - 17:1:0;18:1:0
PhosphatidylCholine - 16:0:0;16:0:0	PhosphatidylEthanolamine - 16:1:0;22:1:0	PhosphatidylInositol - 18:0:0;18:2:0
PhosphatidylCholine - 16:0:0;18:1:0	PhosphatidylEthanolamine - 16:1:0;22:3:0	PhosphatidylInositol - 18:0:0;18:3:0
PhosphatidylCholine - 16:1:0;16:1:0	PhosphatidylEthanolamine - 16:2:0;18:0:0	PhosphatidylInositol - 18:0:0;19:2:0
PhosphatidylCholine - 16:1:0;18:1:0	PhosphatidylEthanolamine - 16:2:0;18:1:0	PhosphatidylInositol - 18:0:0;19:3:0
PhosphatidylCholine - 16:1:0;18:2:0	PhosphatidylEthanolamine - 18:0:0;18:2:0	PhosphatidylInositol - 18:0:0;20:2:0
PhosphatidylCholine - 16:2:0;18:0:0	PhosphatidylEthanolamine - 18:1:0;18:2:0	PhosphatidylInositol - 18:0:0;20:3:0
PhosphatidylCholine - 17:1:0;22:5:0	PhosphatidylEthanolamine - 18:1:0;20:0:0	PhosphatidylInositol - 18:0:0;21:3:0
PhosphatidylCholine - 18:0:0;20:3:0	PhosphatidylEthanolamine Ether - 16:0:0;18:2:0	PhosphatidylInositol - 18:1:0;18:2:0
PhosphatidylCholine Ether - 16:0:0;16:2:0	PhosphatidylEthanolamine Ether - 16:0:0;22:1:0	PhosphatidylInositol - 18:1:0;20:2:0
PhosphatidylCholine Ether - 16:0:0;18:2:0	PhosphatidylEthanolamine Ether - 16:1:0;20:3:0	PhosphatidylInositol - 18:1:0;20:3:0
PhosphatidylCholine Ether - 16:0:0;20:2:0	PhosphatidylEthanolamine Ether - 16:1:0;22:2:0	PhosphatidylInositol - 18:1:0;22:3:0
PhosphatidylCholine Ether - 16:0:0;20:3:0	PhosphatidylEthanolamine Ether - 16:2:0;14:0:0	PhosphatidylInositol - 18:2:0;20:2:0
PhosphatidylCholine Ether - 16:0:0;22:2:0	PhosphatidylEthanolamine Ether - 16:2:0;16:1:0	PhosphatidylInositol - 18:2:0;20:3:0
PhosphatidylCholine Ether - 16:0:0;22:3:0	PhosphatidylEthanolamine Ether - 16:2:0;18:0:0	PhosphatidylSerine - 16:1:0;18:0:0
PhosphatidylCholine Ether - 16:1:0;16:1:0	PhosphatidylEthanolamine Ether - 16:2:0;20:1:0	PhosphatidylSerine - 16:1:0;18:1:0
PhosphatidylCholine Ether - 16:1:0;18:2:0	PhosphatidylEthanolamine Ether - 17:0:0;20:3:0	PhosphatidylSerine - 16:1:0;22:0:0
PhosphatidylCholine Ether - 16:1:0;20:2:0	PhosphatidylEthanolamine Ether - 17:0:0;20:4:0	PhosphatidylSerine - 16:1:0;22:2:0
PhosphatidylCholine Ether - 16:1:0;20:3:0	PhosphatidylEthanolamine Ether - 17:1:0;17:1:0	PhosphatidylSerine - 16:1:0;24:0:0
PhosphatidylCholine Ether - 16:1:0;20:4:0	PhosphatidylEthanolamine Ether - 17:2:0;18:0:0	PhosphatidylSerine - 16:1:0;24:2:0
PhosphatidylCholine Ether - 16:1:0;22:3:0	PhosphatidylEthanolamine Ether - 18:1:0;18:3:0	PhosphatidylSerine - 18:0:0;19:1:0
PhosphatidylCholine Ether - 17:0:0;16:2:0	PhosphatidylEthanolamine Ether - 18:2:0;14:0:0	PhosphatidylSerine - 18:0:0;22:3:0
PhosphatidylCholine Ether - 17:0:0;18:3:0	PhosphatidylEthanolamine Ether - 18:2:0;16:1:0	PhosphatidylSerine - 18:0:0;24:1:0
PhosphatidylCholine Ether - 17:1:0;16:1:0	PhosphatidylEthanolamine Ether - 18:2:0;16:2:0	PhosphatidylSerine - 18:1:0;24:2:0
PhosphatidylCholine Ether - 17:1:0;17:1:0	PhosphatidylEthanolamine Ether - 18:2:0;18:3:0	Triacylglycerol - 50:3:0
PhosphatidylCholine Ether - 17:1:0;18:2:0	PhosphatidylGlycerol - 16:0:0;20:3:0	Triacylglycerol - 51:2:0
PhosphatidylCholine Ether - 17:2:0;16:0:0	PhosphatidylGlycerol - 16:1:0;18:0:0	Triacylglycerol - 52:3:0
PhosphatidylCholine Ether - 17:2:0;18:1:0	PhosphatidylGlycerol - 16:1:0;18:1:0	Triacylglycerol - 52:4:0
PhosphatidylCholine Ether - 18:0:0;16:1:0	PhosphatidylGlycerol - 16:1:0;18:2:0	Triacylglycerol - 53:3:0
PhosphatidylCholine Ether - 18:0:0;18:2:0	PhosphatidylGlycerol - 16:1:0;20:2:0	Triacylglycerol - 54:3:0
PhosphatidylCholine Ether - 18:0:0;20:3:0	PhosphatidylGlycerol - 16:1:0;20:3:0	Triacylglycerol - 54:4:0
PhosphatidylCholine Ether - 18:0:0;20:6:0	PhosphatidylGlycerol - 16:1:0;22:5:0	Triacylglycerol - 54:5:0
PhosphatidylCholine Ether - 18:1:0;14:0:0	PhosphatidylGlycerol - 17:1:0;18:1:0	Triacylglycerol - 56:2:0
PhosphatidylCholine Ether - 18:1:0;16:1:0	PhosphatidylGlycerol - 18:0:0;18:2:0	Triacylglycerol - 56:5:0
PhosphatidylCholine Ether - 18:1:0;16:2:0	PhosphatidylGlycerol - 18:1:0;18:2:0	
PhosphatidylCholine Ether - 18:1:0;18:2:0	PhosphatidylGlycerol - 18:1:0;20:3:0	

Table S4. Materials and resources.

REAGENT or RESOURCE	SOURCE	IDENTIFIER
**Antibodies**		
Mouse monoclonal anti-Involucrin (SY3 and SY7 clones)	In-house	NA
Rabbit polyclonal anti-cleaved Caspase3 (Asp175)	Cell Signalling Technology	Cat. #9661
Donkey anti-Mouse IgG (H+L) Highly Cross-Adsorbed Secondary Antibody, Alexa Fluor 555	Thermo Fisher Scientific	Cat. #A-31570, RRID:AB_2536180
Donkey anti-Rabbit IgG (H+L) Highly Cross-Adsorbed Secondary Antibody, Alexa Fluor 488	Thermo Fisher Scientific	Cat. #A-21206, RRID:AB_2535792
**Chemicals, Peptides, and Recombinant Proteins**		
DRAQ5	abcam	Cat. #ab108410
C14 Ceramide (d18:1/14:0)	Avanti Polar Lipids	Cat. #860514
C16 Ceramide (d18:1/16:0)	Avanti Polar Lipids	Cat. #860516
C24 Ceramide (d18:1/24:0)	Avanti Polar Lipids	Cat. #860524
C18 Glucosyl(ß) Ceramide (d18:1/18:0)	Avanti Polar Lipids	Cat. #860547
C24:1 Glucosyl(ß) Ceramide (d18:1/24:1(15Z))	Avanti Polar Lipids	Cat. #860549
16:0(2R-OH) Ceramide	Avanti Polar Lipids	Cat. #860815
**Critical Commercial Assays**		
RNeasy mini kit	Qiagen	Cat. #74106
QuantiTect Reverse Transcription Kit	Qiagen	Cat. #205311
Fast SYBR® Green Master Mix	Qiagen	Cat. # 4385614
**Experimental Models: Cell Lines**		
Neonatal primary human keratinocyte line (Strain Km)	In-house	NA
**Oligonucleotides**		
ALDH1A2 primers<br>For – AACAAGGCCCTCACAGTGTC<br>Rev – GACATCTTGAATCCCCCAAA	This paper	NA
ALDH8A1 primers<br>For – CCCAGGCCGAGTCTAAAGA<br>Rev – TGGAGGAAGCGAAGAACCTG	This paper	NA
ATP5B primers<br>For – AGGCTGGTTCAGAGGTGTCT<br>Rev – TGGGCAAACGTAGTAGCAGG	This paper	NA
DHCR7 primers<br>For – ACAGAACCGCATCTCAAGGG<br>Rev – AGCTGTACTGGTCACAAGCC	This paper	NA
ELOVL1 primers<br>For – CCAAGGTCAAGGCCAACTGA<br>Rev – CTGACGGACACTGCCCTAAG	This paper	NA
ELOVL3 primers<br>For - CAATGAAGCTCCAGGCTCTC<br>Rev - AACCATGCAGGTAAGGCAAC	This paper	NA
ELOVL4 primers<br>For – CCGGAATGGTCAAATCTCTCC<br>Rev – ACACCATCATCATCAAGCCTC	This paper	NA

EVPL primers<br>For – TGCAGCACGTGGAGGACTACC<br>Rev – CTGTTGCAGCAGCTCTGTGGGG	This paper	NA
IVL primers<br>For – GCCTCAGCCTTACTGTGAGT<br>Rev – TGTTTCATTTGCTCCTGATGG	This paper	NA
LARGE primers<br>For – GCACATCAGCACTCCCTACA<br>Rev – GGCAAGATCGAGCTGGATGA	This paper	NA
PPAP2A primers<br>For – CAGGGAGCTCTGGTTGCAAT<br>Rev – TGCTCGGATAGTGATTCCCAG	This paper	NA
PPL primers<br>For – GCAGAGTGACCTGGCTCGGCT<br>Rev – GCCGCATCCGCCTCTAGCAC	This paper	NA
RPL13A primers<br>For – AACAGCTCATGAGGCTACGG<br>Rev – AACAATGGAGGAAGGGCAGG	This paper	NA
SBSN primers<br>For – AAGGCCGGATGCCAGTTTAG<br>Rev – TGCTGAATGGCAACCATCAAA	This paper	NA
SLC27A1 primers<br>For – AAATCGGGGAGTTCTACGGC<br>Rev – CAGGATGCGGCTGTTGAAAC	This paper	NA
SMPD4 primers<br>For – GTCCGCACACTCTTTAGGCT<br>Rev – AAGCTGCCGAGGAAGTCATC	This paper	NA
TBP primers<br>For – GTGACCCAGCATCACTGTTTC<br>Rev – GAGCATCTCCAGCACACTCT	This paper	NA
TGM1 primers<br>For – GCACCACACAGACGAGTATGA<br>Rev – GGTGATGCGATCAGAGGATTC	This paper	NA
ZNF750 primers<br>For – GCACAGAATGCCTACCTGCC<br>Rev – CCGTTCACAACATTGAGGCTTACT	This paper	NA
**Software and Algorithms**		
GraphPad Prism 8.0	GraphPad Software	RRID:SCR_002798
Microsoft Office Excel 365	Microsoft	RRID:SCR_016137
R v.3.5.1	http://www.r-project.org	NA

Dataset S1 (separate file). Lipidomics of keratinocyte differentiation results. Lipid amounts are given in picomoles.

Dataset S2 (separate file). siRNA screen of lipid-modifying enzymes results. Numbers indicate the percentage of Involucrin-positive cells.

Dataset S3 (separate file). Lipidomics of knocked down keratinocytes results. Lipid amounts are given in picomoles.
